# Predicting adverse drug reactions of combined medication from heterogeneous pharmacologic databases

**DOI:** 10.1186/s12859-018-2520-8

**Published:** 2018-12-31

**Authors:** Yi Zheng, Hui Peng, Xiaocai Zhang, Zhixun Zhao, Jie Yin, Jinyan Li

**Affiliations:** 1 Advanced Analytics Institute, Faculty of Engineering and Information Technology, University of Technology Sydney, 15 Broadway Ultimo, Sydney, 2007 Australia; 2 Discipline of Business Analytics, The University of Sydney, Darlington, Sydney, 2006 Australia

**Keywords:** Drug-drug-ADRs association prediction, Adverse drug reactions, Combined medication, Pharmacologic databases, Negative sample selection

## Abstract

**Background:**

Early and accurate identification of potential adverse drug reactions (ADRs) for combined medication is vital for public health. Existing methods either rely on expensive wet-lab experiments or detecting existing associations from related records. Thus, they inevitably suffer under-reporting, delays in reporting, and inability to detect ADRs for new and rare drugs. The current application of machine learning methods is severely impeded by the lack of proper drug representation and credible negative samples. Therefore, a method to represent drugs properly and to select credible negative samples becomes vital in applying machine learning methods to this problem.

**Results:**

In this work, we propose a machine learning method to predict ADRs of combined medication from pharmacologic databases by building up highly-credible negative samples (HCNS-ADR). Specifically, we fuse heterogeneous information from different databases and represent each drug as a multi-dimensional vector according to its chemical substructures, target proteins, substituents, and related pathways first. Then, a drug-pair vector is obtained by appending the vector of one drug to the other. Next, we construct a drug-disease-gene network and devise a scoring method to measure the interaction probability of every drug pair via network analysis. Drug pairs with lower interaction probability are preferentially selected as negative samples. Following that, the validated positive samples and the selected credible negative samples are projected into a lower-dimensional space using the principal component analysis. Finally, a classifier is built for each ADR using its positive and negative samples with reduced dimensions. The performance of the proposed method is evaluated on simulative prediction for 1276 ADRs and 1048 drugs, comparing using four machine learning algorithms and with two baseline approaches. Extensive experiments show that the proposed way to represent drugs characterizes drugs accurately. With highly-credible negative samples selected by HCNS-ADR, the four machine learning algorithms achieve significant performance improvements. HCNS-ADR is also shown to be able to predict both known and novel drug-drug-ADR associations, outperforming two other baseline approaches significantly.

**Conclusions:**

The results demonstrate that integration of different drug properties to represent drugs are valuable for ADR prediction of combined medication and the selection of highly-credible negative samples can significantly improve the prediction performance.

**Electronic supplementary material:**

The online version of this article (10.1186/s12859-018-2520-8) contains supplementary material, which is available to authorized users.

## Background and motivation

Drug combined medication refers to the scenario where two or more drugs are taken together or concomitantly [1]. It is very common in therapy and clinical practice [2]. For example, it is estimated that up to 82% Americans take one or more drugs, and 29% take more than four drugs together [3]. Consider all drugs have a small chance of side effects, taking different medications combined inevitably increases the overall risk of adverse drug reactions (ADRs) and introduces the additional danger of interactions between medicines. Drug-drug interactions (DDIs) from combined medication have been reported to account for 30% of all ADRs [4], resulting in significant fatality and morbidity [5, 6]. Consequently, early identification of potential ADRs for combined medication is vital to improve drug safety and prevent medication error.

ADRs of combined medication occur when individually safe drugs interact pharmacokinetically or pharmacodynamically [7]. Currently, two major approaches have been developed for detecting potential ADRs of combined medication: pre-marketing review and post-marketing surveillance. In the pre-marketing review process, in vivo and in vitro assays are employed to test new drugs with existing drugs to identify potential risks [8]. However, it is experimentally infeasible to test every possible interaction between a new drug and all existing drugs; let alone, some DDIs manifest only after multiple periods of exposure [8, 9]. Therefore, post-marketing surveillance becomes important. Most post-marketing surveillance studies focus on predicting ADRs for a single drug or drug-drug interactions only [10–12, 12–17]. However, post-marketing ADR identification of combined medication has not been adequately studied.

Current post-marketing surveillance primarily relies on spontaneous reporting systems (SRSs). Harpaz et al. applied association rule mining to detect ADRs of multi-drugs from 162,744 FAERS (Food and Drug Administration Adverse Event Reporting System) reports [2]. They successfully obtained 1,167 multi-drug ADRs associations, demonstrating the feasibility to detect ADRs of multi-drugs from SRSs using conventional data mining tools. Thakrar et al. investigated a multiplicative and an additive statistical model to detect ADRs of drug pairs from FAERS [18]. They validated the two models on 4 known and 4 unknown drug-drug-ADR associations (DDAAs), showing that all 8 DDAAs were successfully predicted by both models. Although SRSs are proved to be a useful data source to detect DDAAs. However, they suffer from common limitations such as high under-reporting ratio, duplicate reports, and delays in reporting, making them unable to detect ADRs of novel or rare drugs.

With the increasing use of electronic health records (EHRs) in hospital and for research, researchers have also attempted to detect ADRs of combined medication from EHRs. For example, Banda et al. investigated the feasibility of prioritizing DDAAs derived from EHRs using four different information sources [7]. They first filtered out known DDAAs found in public databases such as Drugs.com [19] and DrugBank [20] from the candidate DDAA list, and then derived a ranking score measured from the remaining sources. Finally, candidate DDAAs with the highest scores were predicted as identified associations and used for further validation. Iyer et al. used standard methods that measure the disproportionality of the mention of adverse reactions to detect DDAAs from 50 million clinical notes [4]. They showed that using clinical notes their method achieved as good performance as established methods on SRSs (i.e., FARES). In reality, EHRs often have restricted access, because they are always privately owned by health organizations (e.g., hospitals), available only to their cooperated research groups.

On the other hand, social media provides a new channel for people to share experiences and seek for help online. A recent survey shows that 72% of Internet users went online to seek health information [3, 21]. Several attempts have been made to detect DDAAs from social media. For example, White et al. performed a large-scale study on Web search logs to detect a specific DDAA, i.e., paroxetine-pravastatin-hyperglycemia association [22]. Yang et al. proposed to discover drug-drug interactions and DDAAs from MedHelp.org [23], a popular online health community [3]. They first built a heterogeneous healthcare network comprising entities (e.g., drugs, ADRs) extracted from the posts, and then learned a logistic regression classifier using features derived from nodes, links, and triads in the network to predict the occurrences of DDAAs, which achieved satisfactory results. However, social media text is often very noisy, phrased using colloquial language, and potentially contains inaccurate information, making it extremely difficult to extract mentions of drugs and ADRs and to establish their associations when context information (i.e., patient condition) is missing. This has significantly limited the accuracy and reliability of DDAA detection.

To overcome aforementioned limitations, in this work, we explore the use of multiple pharmacologic databases as a new data source to identify ADRs of combined medication. Compared with the above three data sources, pharmacologic databases provide rich useful resources for identifying ADRs of combined medication, owing to the openness, high data quality, and coverage for both novel and rare drugs. Nevertheless, the application of machine learning methods on pharmacologic databases is severely impeded by the lack of proper drug representation and credible negative samples. This is attributed to several reasons. First, each phamacologic database contains different types of data and different aspects of information in relation to drugs, diseases, and ADRs. Such scattered information populates over different databases, lacking an appropriate drug representation to establish the associations between drugs and ADRs. Second, pharmacologic databases, like Twosides databases [24], define a set of positive samples, namely known drug-drug-ADR associations included in the database. However, they often do not contain any explicit set of negative samples—drug-drug-ADRs that have no associations—and this poses a major difficulty in the use of machine learning methods to learn an optimal decision boundary. Therefore, there is a strong need to generate credible negative samples for accurately predicting potential ADRs of combined medication.

In this paper, we propose a method called HCNS-ADR to predict adverse drug reactions of combined medication using credible negative samples selected from pharmacologic databases. We formulate the detection of DDAAs as a binary classification task, where the drug pairs are taken as inputs and ADRs as labels. We fuse heterogeneous information from multiple pharmacologic databases and represent each drug pair as a multi-dimensional vector using their chemical structures, target proteins, substituents, and enriched pathways. To select negative samples, we construct a drug-disease-gene tripartite network and design a scoring method to prioritize interacting drugs through cross-disease analysis on the network. Drug pairs with lower interaction probabilities are preferentially selected as negative samples. Following that, PCA (principal component analysis) [25] is performed on the raw drug-pair vectors to reduce the dimensionality. Finally, we build a supervised classifier for each ADR and evaluate the predication performance with the ADR’s validated positive samples and selected negative samples, demonstrating significant performance gains over baseline methods.

Our work advances the current research as follows: (i) We represent drugs as multi-dimensional vectors according to their heterogeneous features, which makes it possible to employ advanced machine learning methods for predictions; (ii) We design a scoring method on a drug-disease-gene tripartite network to prioritize interacting drugs, paving a way to select credible negative samples for DDAA prediction; (iii) Finally, we learn a high-performance machine learning model for DDAA prediction, providing a list of highly-credible negative drug pairs which can assist the research community in identifying new DDAAs (available in Additional file 1).

## Methods

### Data resources

In this work, we focus on drugs from DrugBank [26], a comprehensive drug database. The drug chemical structures, drug target proteins, and drug substituents are extracted from DrugBank. Since the validated drug target proteins in DrugBank are quite sparse, we also integrate the target information from DrugCentral [27], an open-access online drug compendium. The association data, including drug-gene (Homo sapiens), drug-disease, drug-pathway, and disease-gene associations are retrieved from the CTD (comparative toxicogenomics database) [28]. The drug-drug-ADR associations are downloaded from the Tatonetti Lab [29] (Twosides databases). The integration of the above data finally produces 1048 drugs, 1276 ADRs, and 1,155,754 DDAAs. Information of all researched drugs and ADRs is available in Additional file 2. The disease-gene associations and drug-drug-ADR associations are included in Additional files 3 and 4 respectively. All the above data and the source codes are included in Additional file 5. 
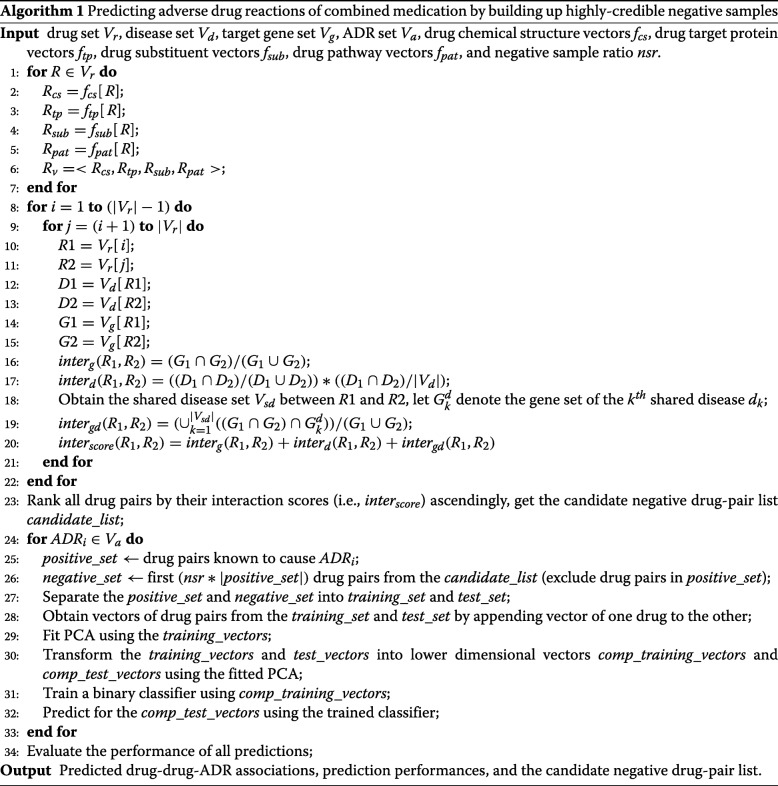


### Proposed method

**Fig. 1:**
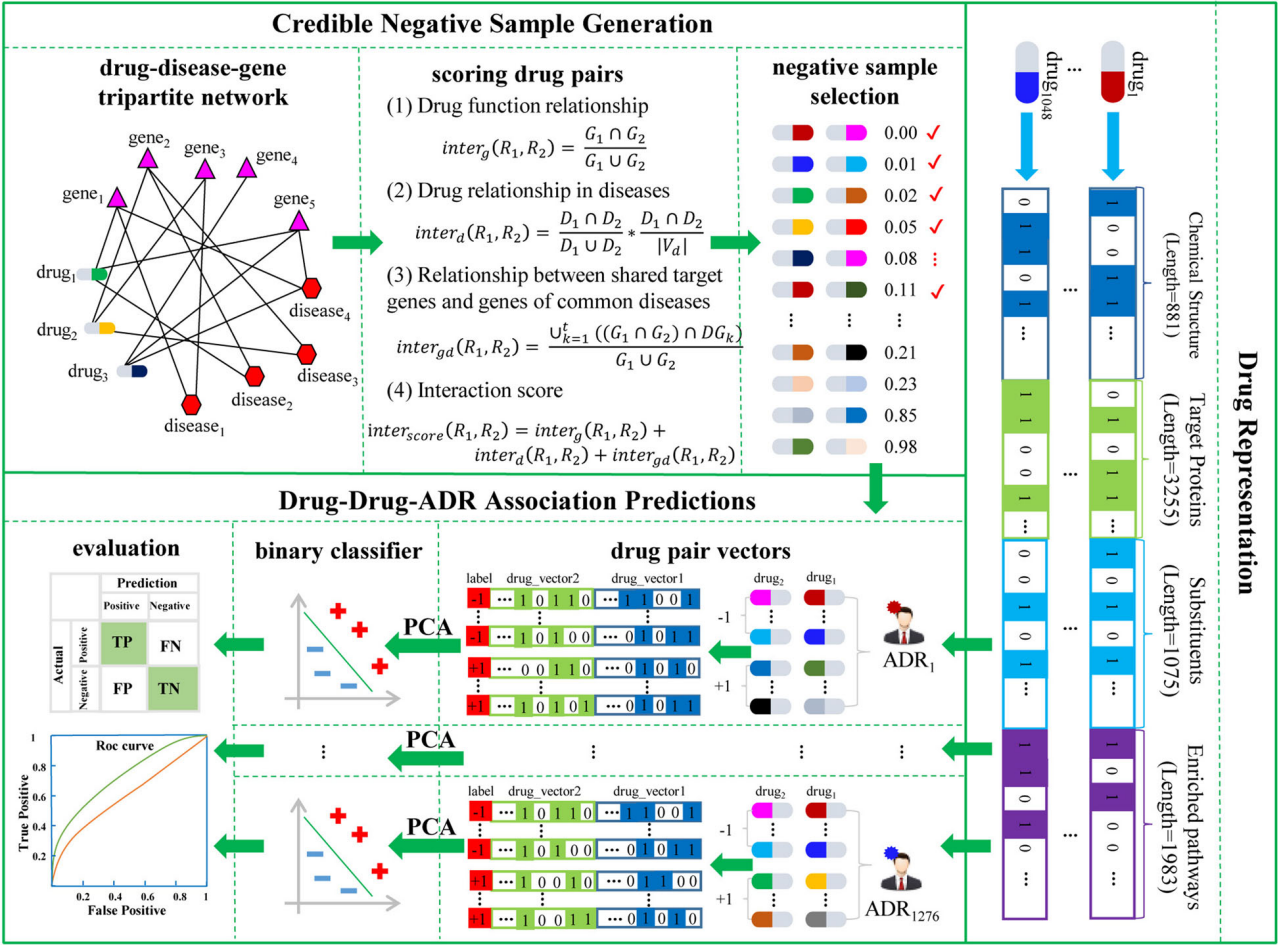
The framework of HCNS-ADR. It consists of three components: drug representation, credible negative sample generation, and drug-drug-ADR association prediction

We cast the prediction of drug-drug-ADR associations into a binary classification task, where inputs are vectors of drug pairs and labels are ADRs. We represent each drug as a multi-dimensional vector using their heterogeneous features. To address the lack of negative samples in the binary classification task, we design a method to select credible negative samples based on a drug-disease-gene tripartite network constructed in our study. The specific process is detailed as pseudo codes in Algorithm 1 and the framework is outlined in Fig. 1. The framework consists of three components, namely drug representation, credible negative sample generation, and drug-drug-ADR association prediction.

#### Drug representation

We represent each drug as a feature vector using its chemical structures, target proteins, substituents, and enriched pathways. Specifically, we use the PubChem fingerprint, which corresponds to 881 substructures defined in the PubChem database [30], to encode the drug chemical structure. Each drug is represented as an 881-bit binary profile, whose elements denote the absence/presence of the corresponding PubChem substructure by 0/1. We obtain 3255 unique drug target proteins by merging target proteins of all drugs. Then each drug can be represented as a 3255 dimensional vector whose elements encode for the absence/presence of the corresponding target proteins by 0/1. Analogously, each drug is represented as a 1075 and 1983 dimensional vector according to its substituents and enriched pathways. Finally, we represent each drug as a single vector by concatenating its four separate vectors. This produces a 7194 (881+3255+1075+1983) dimensional vector for each drug.

#### Credible negative sample generation

In this work, we are interested in predicting adverse drug reactions of combined medication (pair-wise drug medication in particular). These adverse drug reactions can not be attributed to either drug alone, occurring only when two drugs are taken together or concomitantly. Known drug-drug-ADR associations can serve as positive samples for prediction. However, there is a lack of explicit negative samples, namely drug-drug-ADRs that are confirmed to have no associations, making it difficult to directly apply machine learning methods to solve the prediction problem.

Since ADRs of combined medication are caused by pharmacokinetic or pharmacodynamic interactions between drugs [7], it is reasonable to hypothesize a drug pair with lower interaction probabilities are less likely to cause any ADRs. Base on this hypothesis, we explore to use drug pairs with lower interaction probabilities as negative samples for building a classifier. We propose a method to measure the interaction probability of a drug pair according to the following observations: (i) interacting drugs prefer to share the same target genes; (ii) interacting drugs are more likely to associate with a same set of diseases; (iii) the common target genes of two drugs tend to be the common disease genes of their associated diseases.

To explicitly quantify the interaction probability of a drug pair, we first construct a drug-disease-gene (RDG) tripartite network *rdg*=(*V*_*r*_∪*V*_*d*_∪*V*_*g*_,*E*), where *V*_*r*_, *V*_*d*_, *V*_*g*_ is a set of drugs, diseases, and genes, respectively; *E* is the set of associations among them. Given a drug pair *R*_1_ and *R*_2_ (*R*_1_,*R*_2_∈*V*_*r*_), we denote their gene sets as *G*_1_={*g*_11_,*g*_12_,...,*g*_1*i*_,*g*_1*x*_} and *G*_2_={*g*_21_,*g*_22_,...,*g*_2*j*_,*g*_2*y*_}, respectively (*g*_1*i*_,*g*_2*j*_∈*V*_*g*_;(*R*1,*g*_1*i*_),(*R*2,*g*_2*j*_)∈*E*)). Analogously, we represent the disease sets of *R*_1_ and *R*_2_ as *D*_1_={*d*_11_,*d*_12_,...,*d*_1*p*_,*d*_1*m*_} and *D*_2_={*d*_21_,*d*_22_,...,*d*_2*q*_,*d*_2*n*_} respectively (*d*_1*p*_,*d*_2*q*_∈*V*_*g*_;(*D*1,*d*_1*p*_),(*D*2,*d*_2*q*_)∈*E*)). Then the associated genes of a disease *d*_*s*_ can be denoted as *ns*={*g*_1_,*g*_2_,...,*g*_*k*_,...,*g*_*l*_}.

We quantify (i) the function relationship between a drug pair; (ii) the drug therapeutic relationship in different diseases; (iii) the relationship between the shared genes of two drugs and the common disease genes of their associated diseases:

- Drug function relationship. The function relationship between drug *R*_1_ and drug *R*_2_ is measured as the proportion of their shared target genes.
  1 $$ inter_{g}(R_{1}, R_{2}) = \frac{G_{1}\cap G_{2}}{G_{1} \cup G_{2}}.  $$
- Drug therapeutic relationship in diseases. The idea is that drugs have higher overlapping ratio in associated diseases are more likely to interact. Besides, the more diseases two drugs share, the more likely they will interact. The overlapping ratio of associated diseases is measured by dividing the shared disease number by the number of their union diseases. The shared diseases are measured as the percentage of the shared diseases comparing to all diseases in RDG. The two factors are combined to measure drug therapeutic relationship as follows:
  2 $$ inter_{d}(R_{1}, R_{2}) = \frac{D_{1}\cap D_{2}}{D_{1} \cup D_{2}}*\frac{D_{1}\cap D_{2}}{|V_{d}|}.  $$
- Relationship between shared target genes of drug *R*_1_ and drug *R*_2_ and genes of their common diseases. The idea is that interacted drugs always associate with common disease genes which contribute to the disease development.
  3 $$ inter_{gd}(R_{1}, R_{2}) = \frac{\cup_{k=1}^{t} ((G_{1}\cap G_{2}) \cap DG_{k})}{G_{1} \cap G_{2}},  $$

where *DG*_*k*_ is the gene set of the *k*^*th*^ shared disease *d*_*k*_, and *t* is the total number of common diseases between *R*_1_ and *R*_2_.

By integrating the three utilities above, we define the score for calculating the interaction probability of the drug pair *R*_1_ and *R*_2_ as follows:

4 $$ \begin{array}{l} inter_{score}(R_{1}, R_{2}) = inter_{g}(R_{1}, R_{2}) + \\inter_{d}(R_{1}, R_{2}) + inter_{gd}(R_{1}, R_{2}). \end{array}  $$

We rank all candidate drug pairs (548,628) in an ascending order of their interaction scores (i.e., *inter*_*score*_). Drugs from a drug pair with a lower position in the rank are less likely to interact with each other. Therefore, we selectively choose drug pairs with lower positions as negative samples for DDAA prediction.

#### Drug-drug-ADR association prediction

We build a binary classifier for each ADR. Drug pairs known to cause the ADR are used as positive samples directly. A corresponding number of negative samples are selected from the above ranked drug-pair list. The specific number is determined by the negative sample ratio, which will be discussed in the “Results and discussions” section. Each drug pair is represented as a vector by concatenating two individual drug vectors. A drug-pair vector has a high dimensionality of 14,388 (7194 + 7194), which incurs a high computational cost to train a classifier. To speed up training, we employ PCA to perform dimension reduction on the drug-pair vectors. Specifically, all training drug-pair vectors are used to fit the PCA first. Then the fitted PCA is used to transform the training and test drug-pair vectors into lower-dimensional vectors. Finally, the resulted vectors of drug pairs are used as inputs to train and validate the binary classifier.

## Results and discussions

### Performance evaluation metrics

5-fold cross-validation is performed to evaluate the prediction performance: (i) drug pairs in the gold standard set are split into five equal-sized subsets; (ii) each subset is used as the test set, and the remaining four subsets are taken as the training set in turn to train the predictive models; (3) the final performance is evaluated on all results over 5-folds. The macro-averaging value of precision, recall, accuracy, F1-score, and AUC (area under the receiver operating characteristic curve) are used as evaluation metrics.

### Parameter optimization

The key factors of HCNS-ADR are the negative sample ratio (*NSR*) and the PCA component number (*PCN*). Therefore, we first perform experiments to obtain the best settings for *NSR* and *PCN*. We employed SVM (support vector machine) in the Python sklearn package with default settings as the classifier. We experiment with the following settings: *NSR*∈{0.5,1,2,3} and *PCN*∈{10,20,30,40,50,80,100,150,200,300,400,500,600,700,800,900,1000}.

**Fig. 2:**
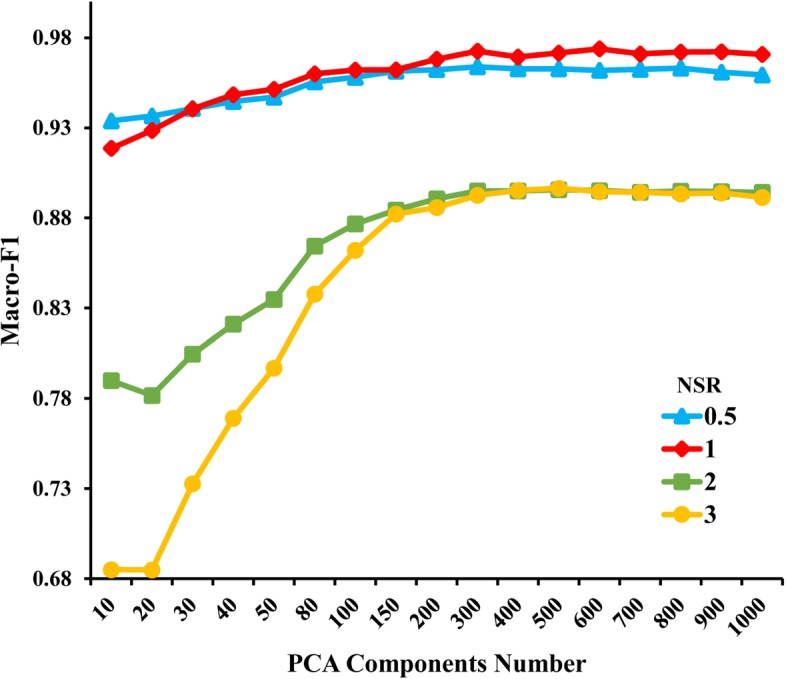
The macro-averaging F1-scores with different PCA component numbers and different negative sample ratios

Figure 2 illustrates the macro-averaging F1-scores for different combinations of *NSR* and *PCN*. It can be observed that, when *PCN*<=300, the macro-F1 increases dramatically with *PCN* for *NSR*=2 (green) and *NSR*=3 (yellow). By contrast, the macro-F1 of *NSR*=0.5 (blue) and *NSR*=1 (red) increases very slightly with *PCN*. And all macro-F1 values plateau when the PCA component number is larger than 300. Similar conclusions can be drawn from the macro-AUC results, as shown in Additional file 6: Figure S1. Besides, *NSR*=1 slightly outperforms *NSR*=0.5, and both of them significantly outperform *NSR*=2 and *NSR*=3. Based on the above observation and considering the time-cost (computational time increases with *PCN*), we set *NSR*=1 and *PCN*=300 for HCNS-ADR in the following experiments.

### Evaluation on classic classifiers

To demonstrate the superior performance of HCNS-ADR and its efficacy of credible negative sample generation, we first conduct experiments to compare four classic classifiers learned based on randomly generated negative samples (RGNS). The randomly generated samples are produced by randomly sampling drug pairs which are not in the positive samples. Except for the way to generate negative samples, other details of RGNS are the same as HCNS-ADR. To avoid bias, RGNS is repeated 5 times and the average results are used for the final evaluation. The four classic classifiers are KNN (k-nearest neighbors), SVM, Random Forest and Logistic Regression. All classifiers are run using Python 2.7.13 (sklearn) with the default settings.

Table 1 shows the evaluation indices of the above four classifiers. We can see that all classifiers achieve significant performance gains using negative samples generated by HCNS-ADR in comparison to their RGNS counterparts using randomly generated samples. For example, as compared to RNGS, HCNS-ADR achieves an improvement of 5.07%, 3.70%, 6.49% and 19.75%, on macro-averaging AUC, and 9.65%, 8.48%, 7.09% and 17.79% on the macro-averaging F1, for SVM, Logistic Regression, KNN, and Random Forest, respectively.

**Table 1:** Macro-averaging AUC, *F*1-score, precision, recall and accuracy of four typical classifiers based on negative samples selected by HCNS-ADR and RGNS

Classifier	Negative Samples	Macro_AUC	Macro_F1	Macro_Precision	Macro_Recall	Macro_Accuracy
SVM	HCNS-ADR	0.994	0.973	0.985	0.963	0.975
SVM	RGNS	0.946	0.887	0.896	0.888	0.893
Logistic regression	HCNS-ADR	0.998	0.980	0.991	0.971	0.981
Logistic regression	RGNS	0.963	0.903	0.898	0.913	0.905
KNN	HCNS-ADR	0.983	0.920	0.972	0.883	0.936
KNN	RGNS	0.923	0.859	0.850	0.877	0.862
Random forest	HCNS-ADR	0.943	0.840	0.928	0.781	0.861
Random forest	RGNS	0.787	0.713	0.753	0.700	0.717

### Comparison with baseline methods

Lots of methods have been developed to predict ADRs, however, they are for single drug use only. To our knowledge, HCNS-ADR is the first method proposed to predict ADRs for combined medication. To further confirm the superiority of HCNS-ADR, we compare it with two baseline methods, namely random assignment and one-class SVM.

**Random Assignment (Random).** To show how difficult the prediction problem is, we use a random assignment method as one baseline, where the label is randomly assigned to a drug pair according to the probability of their ratios. For instance, if the ratio of label “1” in the training set is 50%, we randomly assign “1” to 50% samples in the test set. The other 50% samples in the test set are then assigned with “0”. For this method, the prediction is made at random.

**One-class SVM (ocSVM).** One-class SVM is a one-class learning technique which is widely used in the scenario where there are only positive samples, while negative samples are hard to acquire [31]. It has achieved remarkable performance in a few fields, such as image retrieval, and anomaly detection [32, 33]. One-class SVM is trained with validated positive samples only. In this work, we report evaluation results of ocSVM on two sets of test data: one with negative samples selected randomly (ocSVM_Random), and the other with negative samples generated according to our screen list (ocSVM_Screen). The negative sample ratios of both methods are set as 1.

**Fig. 3:**
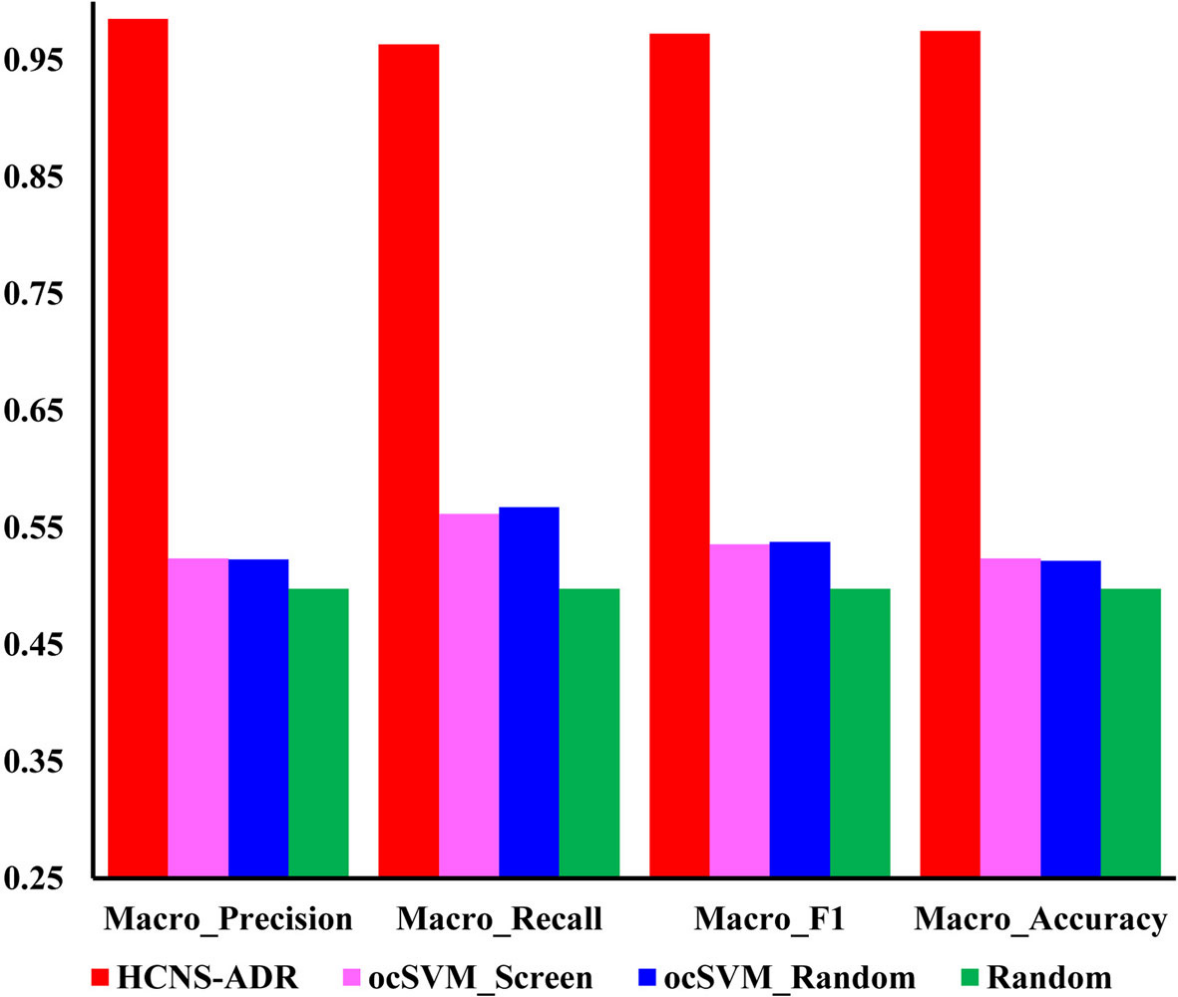
The macro-averaging precision, recall, *F*1 and accuracy of HCNS-ADR and other three comparison methods

To avoid bias, the method ocSVM_Random and Random are repeated 5 times and the average results are used for the final evaluation. The performances achieved by these predictive methods are shown in Fig. 3. Clearly, HCNS-ADR remarkably outperform the other three methods on all evaluation indices. While ocSVM_Screen and ocSVM_Random perform slightly better than the random assignment method, they have comparable prediction results on two test data. It suggests that, with positive samples only, ocSVM is unable to learn an accurate decision boundary for the drug-drug-ADRs association prediction problem. This necessitates the generation of credible negative samples by our proposed method to achieve satisfactory prediction results.

### Predicted adverse drug reactions for the drug pair “Albuterol-Zolpidem”: a case study

After confirming the superior performance of our method, we build 1,276 SVM classifiers using the corresponding validated positive samples and selected negative samples to predict potential ADRs for any drug-pairs. The negative sample ratio is set as 1. Here, we report the prediction results for the drug pair “Albuterol-Zolpidem” as a case study.

**Fig. 4:**
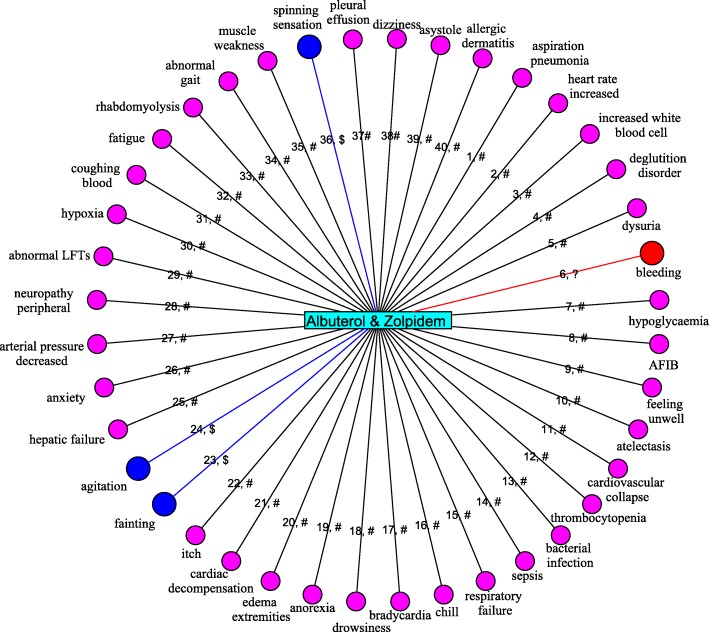
The top 40 ADRs which are predicted to be associated with the drug pair “Albuterol-Zolpidem”. Labels on the edges illustrate the rank of predicted association and the confirmation types. “#” denotes the relation is known in the Tatonetti Lab dataset, “$” means the relation is the common ADRs of the drug pair, “?” indicates there are no evidence for the relation

Like other data mining results, it is unrealistic to expect every highly ranked ADRs valuable to domain experts [6]. Therefore, we shortlist the top 40 ADRs according to their prediction scores, as shown in Fig. 4. The UMLS names of ADRs are labeled on the circle, and their ranks and confirmation types are labeled on the edges. “#” denotes the relation is known in the Tatonetti Lab dataset, “$” means the relation is the common ADRs of the drug pair, and “?” indicates there are no evidences for the relation.

It can be observed that 36 out of the top 40 ADRs are known in the Tatonetti Lab dataset. The other 4 ADRs are newly predicted ADRs for the combined medication “Albuterol-Zolpidem”. We further confirm the newly predicted associations from other data sources; 3 out of them are indeed confirmed as common ADRs of Albuterol and Zolpidem. More specifically, fainting and spinning sensation are found to be two common ADRs of Albuterol and Zolpidem in Drugs.com [19], and agitation is found in SIDER [34], a comprehensive drug side-effect database.

To further demonstrate HCNS-ADR’s ability to predict new DDAAs, we also investigate the ADR ranked list from top-41 to top-50. Among others, acute respiratory distress syndrome, hive, arterial pressure NOS increased, loss of consciousness, ascites and pulmonary arrest are newley predicted ADRs of Albuterol and Zolpidem, requiring further validation. These newly predicted associations provide valuable information to domain experts. In summary, this case study confirms that HCNS-ADR is able to predict both known and novel drug-drug-ADR associations.

## Conclusions

In this work, we propose a machine learning method to predict ADRs of combined medication from pharmacologic databases. To effectively apply machine learning techniques to this prediction problem, we formulate it into a binary classification task, where inputs are vectors of drug pairs and labels are ADRs. We overcome two impediments for the application of machine learning techniques, namely the lack of proper drug representation and credible negative samples. As each pharmacologic database contains different types of data, we leverage heterogeneous information from multiple databases to form an effective drug representation; each drug is represented as a multi-dimensional vector using its chemical substructures, target proteins, substituents, and related pathways. To generate negative samples, we hypothesize that a drug pair with lower interaction probabilities are less likely to cause any ADRs. Therefore, we measure the interaction probability of each drug-pair via analyzing the constructed drug-disease-gene network, and preferentially select drug pairs with lower interaction probabilities as credible negative samples. After performing dimension reduction, we build a classifier for each ADR using its validated positive samples and selected negative samples. The originality of the proposed method lies in formulating DDAA prediction into a binary classification task, in characterizing drugs using their multi-features, in selecting credible negative samples through constructing a comprehensive drug-disease-gene network, and in building predictive models for drug-drug-ADR associations prediction. Both the evaluation on four classic classifiers and comparison experiments with two baseline methods demonstrate the superior performance of the proposed method. Besides, the case study about the drug pair “Albuterol-Zolpidem” confirms that HCNS-ADR is capable to predict both existing and novel drug-drug-ADR associations.

HCNS-ADR is useful in various aspects. First, it can guide the drug development, determine drug molecules should be dropped or kept for further study, and largely reduce the time and financial cost. Besides, early warnings about potential ADRs when drugs are medicated together could be given to pharmacists, doctors, and patients. In this work, only four drug features are employed to represent the drugs. It is worth pointing out that more drug features, e.g., drug indications, can be integrated to characterize drugs. Therefore, we will investigate the impact of different drug features on the DDAA prediction in the near future.

## Additional files

Additional file 1 ∙ Table S1: Drug pairs are sorted in a descending order of their interaction scores.
∙ Table S2: Drug pairs are sorted ascendingly according to their interaction scores. (XLSX 25,647 kb)

Additional file 2 ∙ Table S3: List of 1048 drugs studied in this work. DrugBank ID, Mesh ID, drug names, drug SMILES strings, drug target proteins, substituents, enriched pathways, associated CTD diseases, and associated CTD genes are included as well.
∙ Table S4: List of 1276 adverse drug reaction terms studied in this work. UMLS ID, UMLS name and positive drug pair number are also included. (XLSX 2644 kb)

Additional file 3 The disease-gene associations. This file contains the researched diseases and their associated genes extracted from CTD. (CSV 7229 kb)

Additional file 4 1,155,755 known drug-drug-ADR associations which are extracted from the Tatonetti Lab. Drugs are labeled using their DrugBank IDs, and ADRs are labeled with their UMLS IDs. (CSV 29,346 kb)

Additional file 5 Supplementary codes and data. The Python codes of HCNS-ADR and the source data. (ZIP 10,129 kb)

Additional file 6 ∙ Figure S1: The macro-averaging AUCs with different PCA component number and different negative sample ratios. (PDF 29 kb)

## Abbreviations

ADRs: Adverse drug reactions
AUC: Area under the receiver operating characteristic curve
DDIs: Drug-drug interactions
DDAAs: Drug-drug-ADR associations
EHRs: Electronic health records
FAERS: Food and drug administration adverse event reporting system
KNN: K-nearest neighbor
NSR: Negative sample ratio
ocSVM: One-class SVM
PCA: Principal component analysis
PCN: PCA component number
PBs: Pharmacological databases
SRSs: Spontaneous reporting systems
SVM: Support vector machine

## References

[1] Crits-Christoph P, Newman MG, Rickels K, Gallop R, Gibbons MBC, Hamilton JL, Ring-Kurtz S, Pastva AM (2011). Combined medication and cognitive therapy for generalized anxiety disorder. J Anxiety Disord.

[2] Harpaz R, Chase HS, Friedman C. Mining multi-item drug adverse effect associations in spontaneous reporting systems. In: BMC Bioinformatics: 2010. p. 7. BioMed Central. 10.1186/1471-2105-11-S9-S7 PMC2967748 21044365

[3] Yang H, Yang CC (2016). Discovering drug-drug interactions and associated adverse drug reactions with triad prediction in heterogeneous healthcare networks. 2016 IEEE International Conference on Healthcare Informatics (ICHI).

[4] Iyer SV, Harpaz R, LePendu P, Bauer-Mehren A, Shah NH (2013). Mining clinical text for signals of adverse drug-drug interactions. J Am Med Inform Assoc.

[5] Lazarou J, Pomeranz BH, Corey PN (2012). Incidence of adverse drug reactions in hospitalized patients. JAMA.

[6] Zheng Y, Lan C, Peng H, Li J (2016). Using constrained information entropy to detect rare adverse drug reactions from medical forums. 2016 IEEE 38th Annual International Conference on Engineering in Medicine and Biology Society (EMBC).

[7] Banda JM, Callahan A, Winnenburg R, Strasberg HR, Cami A, Reis BY, Vilar S, Hripcsak G, Dumontier M, Shah NH (2016). Feasibility of prioritizing drug–drug-event associations found in electronic health records. Drug Saf.

[8] Zhang L, Zhang YD, Zhao P, Huang S-M (2009). Predicting drug–drug interactions: an fda perspective. The AAPS J.

[9] Triaridis S, Tsiropoulos G, Rachovitsas D, Psillas G, Vital V (2009). Spontaneous haematoma of the pharynx due to a rare drug interaction. Hippokratia.

[10] Liu J, Zhao S, Zhang X (2016). An ensemble method for extracting adverse drug events from social media. Artif Intell Med.

[11] Zhang W, Chen Y, Tu S, Liu F, Qu Q (2016). Drug side effect prediction through linear neighborhoods and multiple data source integration. IEEE International Conference on Bioinformatics and Biomedicine (BIBM).

[12] Yang CC, Jiang L, Yang H, Tang X (2012). Detecting signals of adverse drug reactions from health consumer contributed content in social media. Proceedings of ACM SIGKDD Workshop on Health Informatics.

[13] Liu X, Chen H (2013). Azdrugminer: an information extraction system for mining patient-reported adverse drug events in online patient forums. Smart Health.

[14] Zheng Y, Ghosh S, Li J (2017). An optimized drug similarity framework for side-effect prediction. Comput Cardiol.

[15] Leaman R, Wojtulewicz L, Sullivan R, Skariah A, Yang J, Gonzalez G (2010). Towards internet-age pharmacovigilance: extracting adverse drug reactions from user posts to health-related social networks. Proceedings of the 2010 Workshop on Biomedical Natural Language Processing.

[16] Yamanishi Y, Pauwels E, Kotera M (2012). Drug side-effect prediction based on the integration of chemical and biological spaces. J Chem Inf Model.

[17] Hammann F, Gutmann H, Vogt N, Helma C, Drewe J (2010). Prediction of adverse drug reactions using decision tree modeling. Clin Pharmacol Ther.

[18] Thakrar BT, Grundschober SB, Doessegger L (2007). Detecting signals of drug–drug interactions in a spontaneous reports database. Br J Clin Pharmacol.

[19] Wikipedia contributors. Drugs.com —Wikipedia The Free Encyclopedia. 2018. https://en.wikipedia.org/w/index.php?title=Drugs.com&oldid=839918274 Accessed 15 May 2018.

[20] Law V, Knox C, Djoumbou Y, Jewison T, Guo AC, Liu Y, Maciejewski A, Arndt D, Wilson M, Neveu V (2013). Drugbank 4.0: shedding new light on drug metabolism. Nucleic Acids Res.

[21] White RW, Wang S, Pant A, Harpaz R, Shukla P, Sun W, DuMouchel W, Horvitz E (2016). Early identification of adverse drug reactions from search log data. J Biomed Inform.

[22] White RW, Tatonetti NP, Shah NH, Altman RB, Horvitz E (2013). Web-scale pharmacovigilance: listening to signals from the crowd. J Am Med Inform Assoc.

[23] MedHelp.org. Medical Information, forums and communities. https://www.medhelp.org 2 May 2018.

[24] Tatonetti NP, Fernald GH, Altman RB (2011). A novel signal detection algorithm for identifying hidden drug-drug interactions in adverse event reports. J Am Med Inform Assoc.

[25] Bro R, Smilde AK (2014). Principal component analysis. Anal Methods.

[26] Wishart DS, Feunang YD, Guo AC, Lo EJ, Marcu A, Grant JR, Sajed T, Johnson D, Li C, Sayeeda Z (2017). Drugbank 5.0: a major update to the drugbank database for 2018. Nucleic Acids Res.

[27] Ursu O, Holmes J, Knockel J, Bologa CG, Yang JJ, Mathias SL, Nelson SJ, Oprea TI (2017). Drugcentral: online drug compendium. Nucleic Acids Res.

[28] Davis AP, Grondin CJ, Johnson RJ, Sciaky D, King BL, McMorran R, Wiegers J, Wiegers TC, Mattingly CJ (2016). The comparative toxicogenomics database: update 2017. Nucleic Acids Res.

[29] Tatonetti NP, Patrick PY, Daneshjou R, Altman RB (2012). Data-driven prediction of drug effects and interactions. Sci Transl Med.

[30] Chen B, Wild D, Guha R (2009). Pubchem as a source of polypharmacology. J Chem Inf Model.

[31] Erfani SM, Rajasegarar S, Karunasekera S, Leckie C (2016). High-dimensional and large-scale anomaly detection using a linear one-class svm with deep learning. Pattern Recog.

[32] Chen Y, Zhou XS, Huang TS (2001). One-class svm for learning in image retrieval. 2001 International Conference on Image Processing, 2001. Proceedings.

[33] Li K-L, Huang H-K, Tian S-F, Xu W (2003). Improving one-class svm for anomaly detection. 2003 International Conference on Machine Learning and Cybernetics.

[34] Kuhn M, Letunic I, Jensen LJ, Bork P (2015). The sider database of drugs and side effects. Nucleic Acids Res.

