# Supplementary material for: Predicting adverse drug reactions of combined medication from heterogeneous pharmacologic databases
Source: BMC Bioinformatics. 2018 Dec 31;19(Suppl 19):517. doi: 10.1186/s12859-018-2520-8 (PMC6311930; doi:10.1186/s12859-018-2520-8)
Supplement: Supplementary file 6 — ∙ Figure S1: The macro-averaging AUCs with different PCA component number and different negative sample ratios. (PDF 29 kb) [file 12859_2018_2520_MOESM6_ESM.pdf]

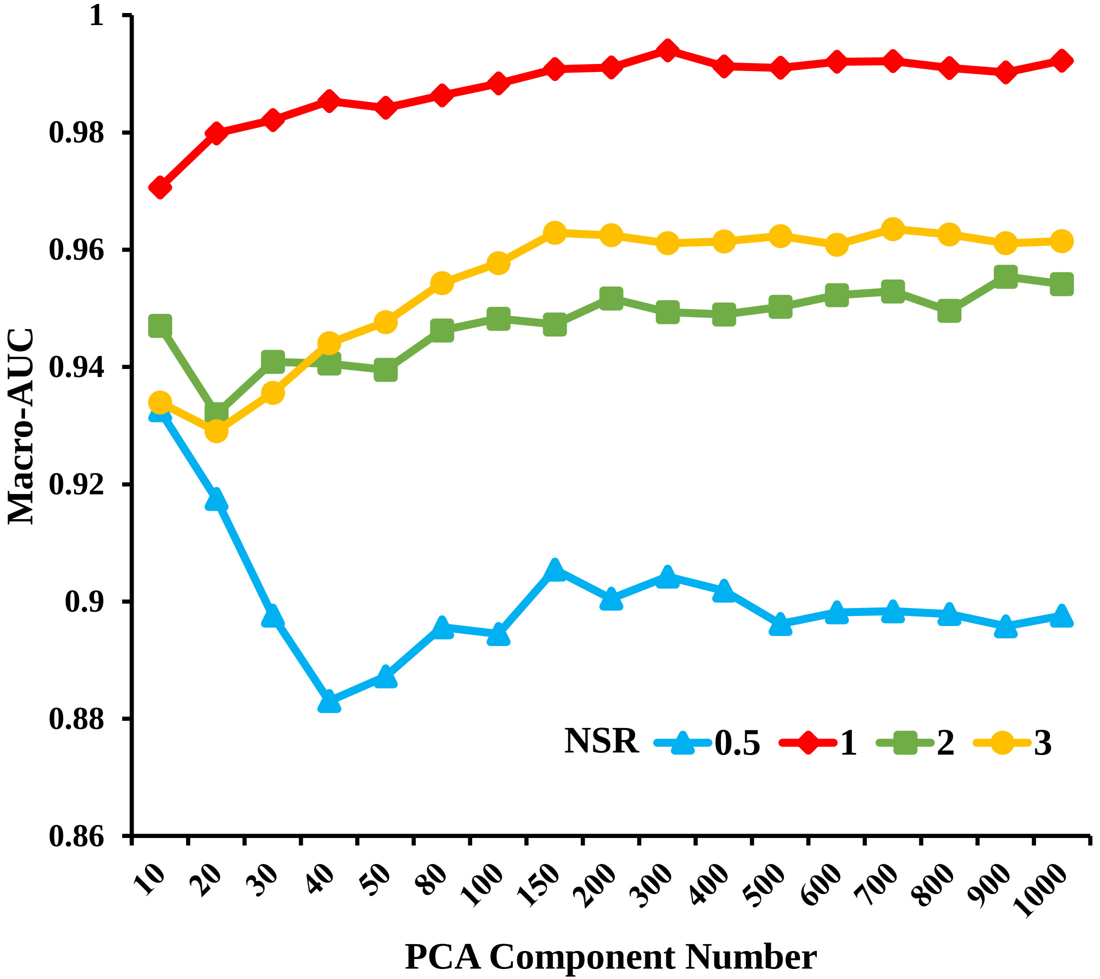

Figure S1. The macro-averaging AUCs with different PCA component number and different negative sample ratios.
